# The Allelic and Phenotypic Frequencies of the ABO and Rh Blood Types in Pregnant Women in Addis Ababa, Ethiopia

**DOI:** 10.1155/bmri/8649988

**Published:** 2025-02-13

**Authors:** Mekdes Wondiye Tedbabe, Dagim Jirata Birri, Takele Taye Desta

**Affiliations:** ^1^Department of Biology, Kotebe University of Education, Addis Ababa, Ethiopia; ^2^Department of Microbial, Cellular and Molecular Biology, Addis Ababa University, Addis Ababa, Ethiopia

**Keywords:** ABO and Rh blood groups, Hardy–Weinberg equilibrium, phenotypic and allelic frequencies, red blood cells' antigens, transfusion medicine

## Abstract

**Background:** ABO–rhesus (Rh) blood testing screens blood types according to the antigenic properties of red blood cells.

**Objective:** This study reports the allelic and phenotypic frequency distribution of the ABO and Rh blood groups in pregnant women who attended antenatal care (ANC) at Zewditu Memorial Hospital in Addis Ababa, Ethiopia, and the likelihood for the occurrence of erythroblastosis fetalis (E. fetalis).

**Methods:** A retrospective study was conducted on pregnant women who attended ANC from 2015 to 2019 and typed for ABO and Rh blood groups. The Hardy–Weinberg equilibrium was used to determine the allelic frequency of ABO and Rh blood types. The likelihood of the occurrence of E. fetalis was computed.

**Results:** Among the 2453 women who had been admitted to ANC, 2407 (98.1%) pregnant women who had been typed for the ABO and Rh blood groups were included in this retrospective study. We found that Type O blood was the most common one (38.9%), while Types A (31.3%), B (23.8%), and AB (6.0%) blood were scored with modest to lower proportions. Among blood group–typed women, 94.2% were Rh-positive. The allelic frequency of O was 0.62, whereas A (0.22) and B (0.16) had modest proportions. The allelic frequency of D was 0.76 and d was 0.24. The likelihood of the occurrence of E. fetalis was 5%. Our findings show that both the ABO (*χ*‐*squared* = 6.1439, *df* = 3, *p* *value* = 0.1048) and the Rh (*χ*‐*squared* = 0.000103, *df* = 1, *p* *value* = 0.9919) blood groups were segregated at the Hardy–Weinberg proportions. Studies need to investigate the evolutionary forces that have made the ABO and Rh blood types segregate at the Hardy–Weinberg proportion.

## 1. Introduction

Blood grouping sorts blood based on the antigenic properties of the red blood cells (RBCs) [1–5]. The ABO blood group consists of two antigens, A and B, on the surface of the RBCs and two reciprocal antibodies (anti-A and anti-B) in the blood plasma (serum). Phenotypically, individuals who are homozygous dominant (DD) or heterozygous (Dd) are rhesus (Rh)-positive, whereas those who are homozygous recessive (dd) are Rh-negative [6]. Blood group antigens play an important role in transfusion medicine, in genetic studies, and in screening for disease susceptibility [7–9]. An Austrian scientist named Karl Landsteiner was the first to discover the ABO blood group system [1, 2]. Nearly 700 antigens have been identified in erythrocytes. The antigens are categorized into 44 blood group systems by the International Society of Blood Transfusion [10]. Among the blood group systems, the ABO and Rh blood types are the most important due to their use in blood transfusion [11]. According to the presence of antigens and agglutination patterns, the ABO blood group is divided into four types: A, B, O [1, 2], and AB [3].

The second-most important blood group type is Rh, which was discovered in the 1940s by Karl Landsteiner and Alexander Weiner [12, 13]. In the Rh blood group system, the Rh antigen, named after the antigen first discovered in Rh monkeys, is found on the surface of the RBC. Individuals who have the RhD antigen are Rh-positive, whereas those who lack it are Rh-negative. The frequencies of ABO and Rh blood groups could vary among populations sharing the same geographic region [14, 15].

For proper management of blood banks and safe blood transfusions, knowledge of the frequency distribution of ABO and Rh blood groups is crucial [8]. Blood transfusion refers to the transfer of blood or blood products from a donor to the recipient [16]. O-negative blood types are safe for everyone. Consequently, individuals with an O-negative blood type are referred to as universal donors. The O-negative blood type is used in emergency cases when there is no time to test the recipient's blood type. On the other side, Type AB blood is a universal blood plasma donor. O is recessive to both the A and B alleles. The AB blood type is codominant and a universal recipient. Individuals who have Rh-positive blood can receive either Rh-positive or Rh-negative blood, but those who are Rh-negative can only receive Rh-negative blood [16].

Identifying the ABO and Rh blood groups is important, especially in transfusion medicine and to treat erythroblastosis fetalis (E. fetalis). Moreover, testing for blood types is vital in the Global South, where blood group screening practices are less common. This study reports the frequency distribution of the ABO and Rh blood groups in pregnant women who attended antenatal care (ANC) at Zewditu Memorial Hospital in Addis Ababa, Ethiopia.

## 2. Materials and Methods

### 2.1. The Study Sites and Population

The study was conducted at Zewditu Memorial Hospital in Addis Ababa, Ethiopia. Among the 12 public-owned hospitals in Addis Ababa, Zewditu Memorial Hospital was selected because it is the closest hospital to Gandhi Memorial Hospital, which specializes in maternity care. As a result, Zewditu Memorial Hospital is an immediate outlet for pregnant women when there is a spillover at Gandhi Memorial Hospital. Pregnant women who visited Zewditu Memorial Hospital for ANC from September 2015 to June 2019 and were typed for the ABO and Rh blood groups were included in this retrospective study.

### 2.2. The Study Designs

The medical histories of 2453 pregnant women who visited Zewditu Memorial Hospital were considered in this study, and out of these, 2407 had been typed for ABO and Rh blood groups. The ABO and Rh blood data were collected from four ANC departments and 10 ANC registration books.

### 2.3. Data Analysis

Inferences were made using the chi-square test and Cramer's *V* test. The data were analyzed using SPSS Version 22 [17] and R [18]. When the *p* value is less than or equal to 0.05, the analysis is deemed significant.

### 2.4. Allelic and Genotypic Frequency Analyses

For the Rh blood group, the allelic frequencies were calculated using Equations (1) and (2), and the genotypic frequencies were calculated using Equations (3)–(5).

The allelic frequency is calculated as follows:

(1) $$\begin{array}{c} f\left(d\right)=\sqrt{\frac{q^{2}}{N}} \end{array}$$

(2) $$\begin{array}{c} f\left(D\right)=1-f\left(d\right) \end{array}$$

The genotypic frequency is calculated as follows:

(3) $$\begin{array}{c} f\left(\text{dd}\right)=q^{2} \end{array}$$

(4) $$\begin{array}{c} f\left(\text{Dd}\right)=2pq \end{array}$$

(5) $$\begin{array}{c} f\left(\text{DD}\right)=p^{2} \end{array}$$

The phenotypic frequency for the biallelic Rh factor with a complete dominance expression pattern was computed as

(6) $$\begin{array}{c} \text{Homozygous recessive individuals}=q^{2} \end{array}$$

(7) $$\begin{array}{c} \text{Homozygous dominant and heterozygous individuals}=p^{2}+2pq \end{array}$$

The Hardy–Weinberg equilibrium for the Rh factor was analyzed using Equation (8).

(8) $$\begin{array}{c} p2+2pq+q2=1 \end{array}$$

For the multiallelic ABO blood group, the allelic frequencies were calculated using Equations (9)–(11), and the genotypic frequencies were calculated using Equations (12)–(17).

(9) $$\begin{array}{c} f\left(O\right)=\sqrt{\frac{q^{2}}{N}} \end{array}$$

The frequency of the A allele was obtained using the quadratic equation (Equation (10)).

(10) $$\begin{array}{c} \frac{-b\pm \sqrt{b^{2}-4ac}}{2a} \end{array}$$

The frequency of the B allele was calculated using Equation (11).

(11) $$\begin{array}{c} f\left(B\right)=1-\left(f\left(A\right)+f\left(B\right)\right) \end{array}$$

The genotypic frequency of ABO blood groups was calculated using Equations (12)–(17).

(12) $$\begin{array}{c} \;f\left(\text{OO}\right)=r^{2} \end{array}$$

(13) $$\begin{array}{c} f\left(\text{AA}\right)=p^{2} \end{array}$$

(14) $$\begin{array}{c} f\left(\text{AO}\right)=2pr \end{array}$$

(15) $$\begin{array}{c} f\left(\text{BB}\right)=q^{2} \end{array}$$

(16) $$\begin{array}{c} f\left(\text{BO}\right)=2qr \end{array}$$

(17) $$\begin{array}{c} f\left(\text{AB}\right)=2pq \end{array}$$

The Hardy–Weinberg equilibrium for the ABO blood group was analyzed using Equation (18).

(18) $$\begin{array}{c} p^{2}+2pq+q^{2}+2pr+2qr+r^{2}=1 \end{array}$$

Phenotypic frequencies were calculated using Equations (19)–(22).

(19) $$\begin{array}{c} \text{Blood group }O=r^{2} \end{array}$$

(20) $$\begin{array}{c} \text{Blood group }A\left(\text{AA and AO}\right)=p^{2}+2pr \end{array}$$

(21) $$\begin{array}{c} \text{Blood group }B\left(\text{BB and BO}\right)=q^{2}+2qr \end{array}$$

(22) $$\begin{array}{c} \text{Blood group AB}=2pq \end{array}$$

The occurrence of E. fetalis was computed using Equation (23).

(23) $$\begin{array}{c} P\left(\text{E}.\text{fetalis}\right)=\left[p\left(\text{DD}\right)*p\left(\text{dd}\right)\right]+\left[\frac{p\left(\text{Dd}\right)*p\left(\text{dd}\right)}{2}\right] \end{array}$$

where *p* is the probability, DD is the homozygote dominant genotype, Dd is the heterozygote, and dd is the homozygote recessive genotype.

## 3. Results

### 3.1. Demographic Characteristics of the Pregnant Women

The demographic structure of the pregnant women who visited Zewditu Memorial Hospital for ANC and who were typed for the ABO and Rh blood groups shows a significant difference in most of the recorded demographic characteristics (Table 1). Few women were below the age of 19.

### 3.2. ABO and Rh Blood Group Frequencies

**Figure 1:**
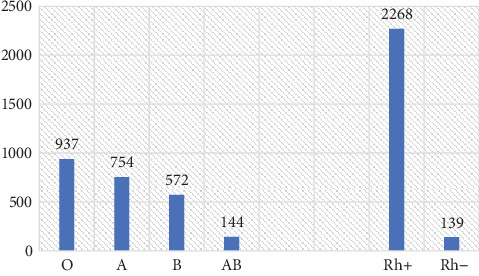
ABO and Rh blood groups of pregnant women who visited Zewditu Memorial Hospital from September 2015 to June 2019.

Type O blood was the most frequent and accounted for 38.9%, followed by A (31.3%), B (23.8%), and AB (6.0%). Among the blood group–typed women, 94.2% were Rh-positive (Figure 1). A statistically significant difference was observed between the proportions of the ABO (*χ*‐*squared* = 576.39, *df* = 3, *p* *value* < 2.2*e* − 16) and Rh (*χ*‐*squared* = 1886.1, *df* = 1, *p* *value* < 2.2*e* − 16) blood types. Our analysis shows that the ABO blood group alleles were segregated at the Hardy–Weinberg equilibrium (*χ*‐*squared* = 6.1439, *df* = 3, *p* *value* = 0.1048). Likewise, for the Rh-factor allelic frequency, the population was at the Hardy–Weinberg equilibrium (*χ*‐*squared* = 0.000103, *df* = 1, *p* *value* = 0.9919).

The result of cross-tabulation for ABO and Rh blood types is presented in Table 2. Cramer's *V* analysis shows that there is a significant association between ABO and Rh blood types (*V* = 0.564, *p* < 0.001).

### 3.3. Allelic and Genotypic Frequencies of ABO and Rh Blood Groups

The allelic frequency of d was 0.24; thus, D was 0.76. The allelic frequency of the O blood type was 0.62, A was 0.22, and B was 0.16. The frequencies of the six genotypic classes of the ABO blood group and the Rh factor are presented in Table 3.

### 3.4. The Combined Genotypic Frequencies of ABO and Rh Blood Groups

The combined genotypic frequencies of the ABO and Rh blood groups are presented in Table 4. The likelihood of E. fetalis in the studied population can be estimated using the sum rule of probability. Accordingly, mating between DD men and dd women, plus half of the mating between Dd men and dd women, would have the chance of creating Rh-positive pregnancies carried by Rh-negative women. According to this data and by assuming similar allelic frequencies in male and female sex groups, the likelihood of the occurrence of the hemolytic reproductive disorder in fetuses and neonates is about 5%.

## 4. Discussions

A small percentage of young women were phenotyped for the ABO–Rh blood group because early marriage is not common in metropolitan Addis Ababa. However, this abhorrent practice is widespread in rural Ethiopia [19]. The data also subtly demonstrates that having children is typically linked to marriage, demonstrating the conservative mindset of this ancient nation's residents who firmly thought that having children should be linked to common-law marriage [20]. The study assessed the frequency distribution pattern of ABO and Rh blood groups of pregnant women in Addis Ababa. The retrospective data was generated from demographically heterogeneous study subjects, corroborating its representativeness. In this study, Type O blood was the most frequent, and Type AB blood was the least. The rarity of Type AB blood is not surprising because it is the product of the joint probabilities of the two minor alleles A and B of the ABO blood group. In line with this finding, several studies conducted on the ABO blood group show that the frequency of Type O blood is the highest [21–24]. However, Type B blood is the most frequent in some regions of India [25], and Type A blood is most frequent in Japan [26] and in some tribal communities of India [21]. Blood group frequency could vary among ethnic groups and even across religious sects [21] and can be varied following environmental gradients [27]. Consanguine marriage instigated by religious dogmas may alter the allelic frequency by introducing homogeneity, which could also hold for blood types.

The high frequency of the O allele is observed regardless of its recessive expression pattern. However, being expressed in a recessive manner does not necessarily reflect the characteristics of a rare allele. Because O may possess a selective advantage, it has a high historical allelic frequency. Undoubtedly, in most parts of the world and likely across the globe, Allele O represents the major allele, and the Sibling A and B alleles represent minor alleles. According to Ségurel et al. [28], our close phylogenetic relatives like chimpanzees, gorillas, orangutans, and gibbons possess a high proportion of Types A and B blood, questioning the ancestral nature of the O allele. However, we need to recall the recessive expression pattern of Allele O, which does not enable it to be expressed in the heterozygote state (AO and BO). On the other hand, by analogy, this tells us that A and B alleles might be ancestral. However, O, which originated from a point mutation bringing about a frameshift mutation [29], is segregating at a higher frequency. This might be attributed to its selective advantage, which partly derives from the production of both A and B antibodies in the blood plasma [30]. It was believed to be ancient human races like the Red Indians of South America, and Eskimos possess a high frequency of Type O blood [27]. Types A and B blood sometimes reshuffle their ranking order of frequency count [31] as O and A do. However, Type AB blood invariably remains the least observed phenotype [31]. Because Type AB blood lacks both Antibodies A and B in the blood plasma, it might have lost some comparative adaptive advantages.

The Rh-positive allele is invariably the most common across the world. Subsequently, the Rh-positive allele represents the major allele. For example, in line with this study, the Rh-positive blood type is the most common one among several communities [21–24]. Consequently, the frequency distribution of the Rh-negative blood type is low. Regardless of the low proportion of Rh-negative blood, it needs due attention to reduce its adverse impact on the reproductive health of fetuses and neonates. Rh-negative blood type is extremely rare in Asia [32]; however, it is relatively common among Caucasian populations [33]. The Rh-negative allele might have arisen later in evolutionary history as a recessive mutation, which is why its frequency is much lower than that of the putative ancestral allele, Rh-positive. Rh-negative blood might have lost its selective advantage, especially regarding reproductive fitness. However, Rh-negative individuals are thought to be resilient to infections [8]. Although the ABO and Rh blood groups are encoded by different genes that are located on different chromosomes, the two blood groups are functionally linked.

E. fetalis accounts for about 3% of infant deaths [34], which is comparable to the likelihood of the incidence of E. fetalis reported in our study for both fetuses and neonates (5%). As it was described in the methodology section (Equation (23)), when an Rh-negative mother is immunized by the Rh-positive RBCs of the fetus, or when this mother is transfused with Rh-positive blood, E. fetalis could occur [35, 36]. However, besides the placental barrier between maternal and fetal blood, the actively growing fetus may, in some instances, produce sufficient blood through the process of extramedullary erythropoiesis in the spleen and liver [36]. Moreover, the strength of the antibody produced by the mother, the gestational stage, and the ability of the fetus to replenish the destroyed RBCs and clear bilirubin determine the impact of E. fetalis [37]. Once the Rh-negative mother was immunized using Rh(D) immune globulin, maternal antibodies to Rh-positive cells were not produced in subsequent pregnancies, making the pregnancies safe [37]. Although rare, anti-Kell E. fetalis is identical to the health disorder that is caused by anti-Rh(D) [38, 39]; therefore, disentangling the two pathophysiologies is essential for the effective treatment of the hemolytic disease of the fetus and neonate.

The Hardy–Weinberg equilibrium analyses exhibit statistically inconsequential differences among observed and expected ABO blood groups (*p* *value* = 0.1048) and Rh factor (*p* *value* = 0.9919) allelic proportions. The Hardy–Weinberg proportions can be attained regardless of nonrandom mating [40] and inherently outbreeding practices in human races. The maintenance at the Hardy–Weinberg proportion encountered regardless of the ABO blood group involves a complex expression pattern involving both complete dominance and codominance expression patterns at a locus level, multiallelism, intricate gene flow, and genetic admixture among human races. However, humans rarely consider blood types while selecting mates; hence, blood group types are little impacted by anthropogenic effects. Moreover, long-term balanced selection could have maintained the alleles at the Hardy–Weinberg proportion. Besides, even these days, a few individuals, especially in the Global South, are aware of their blood types. Often, there is no sex bias in the allelic frequencies of ABO and Rh blood groups [24], which enables to maintain the Hardy–Weinberg equilibrium. Because humans are sexually reproducing organisms and their population size is sufficiently large, these attributes can also contribute to the maintenance of the Hardy–Weinberg proportion. Humans might have relatively experienced neutral selection [41], which could be why they possess various alleles that are segregating at the Hardy–Weinberg proportion.

## 5. Conclusion

The phenotypic frequency distribution of the ABO blood group according to decreasing order of frequency count was O, A, B, and AB. Rh-positive was the most frequent. Both ABO and Rh blood groups were segregated at the Hardy–Weinberg proportion. This is regardless of the practice of nonrandom mating and expansive gene flow in humans. The outcome of this study will contribute its part to the blood transfusion process and take corrective measures on the adverse impact of E. fetalis. The findings of this study need to be corroborated using a large dataset involving study subjects with various demographic structures. Along with blood group typing, future studies need to focus on unraveling the evolutionary history of ABO and Rh blood group alleles and mapping the drivers of ABO and Rh blood group allelic frequency variations.

## Figures and Tables

**Table 1:** Demographic characteristics of the study population.

**Variables**	**Frequency**	**Percent**
Age groups		*χ*‐*squared* = 1737.2, *df* = 5, *p* *value* < 2.2*e* − 16
<19	50	2.0
20–24	607	24.7
25–30	980	40.0
31–34	562	22.9
35–39	224	9.1
40–45	16	0.7
Not recorded	14	0.5
Marital status		*χ*‐*squared* = 6009, *df* = 3, *p* *value* < 2.2*e* − 16
Single	26	1.1
Married	2052	83.7
Divorced	2	0.1
Widowed	2	0.1
Not recorded	371	15.1
Educational status		*χ*‐*squared* = 35.103, *df* = 1, *p* *value* = 3.128*e* − 09
Illiterate	1	0.0
Literate	38	1.6
Not recorded	2412	98.3
Employment status		*χ*‐*squared* = 0.67123, *df* = 1, *p* *value* = 0.4126
Employed	40	1.6
Unemployed	33	1.3
Not recorded	2378	97.0

**Table 2:** Cross-tabulation of frequency counts of ABO and Rh blood groups.

**ABO–Rh blood group**	**Rh blood group**		**Total**
	**Rh ** ^**+**^	**Rh ** ^**−**^	
ABO blood group			
A	705	49	754
B	544	28	572
AB	133	11	144
O	886	51	937
Total	2268	139	2407

**Table 3:** Genotypic frequencies of ABO and Rh blood groups.

**Blood group genotypes**	**Equation**	**Genotypic frequencies**
ABO		
AA	*p* ^2^	0.0484
AO	2*pr*	0.2728
BB	*q* ^2^	0.0256
BO	2*qr*	0.1984
AB	2*pq*	0.0704
OO	*r* ^2^	0.3844
Rh factor		
DD	*p* ^2^	0.5776
Dd	2*pq*	0.3648
dd	*q* ^2^	0.0576

**Table 4:** The combined genotypic frequencies of ABO and Rh blood groups.

**ABO and Rh blood group types**	**Rh blood group types**	
	**Rh ** ^**+**^	**Rh ** ^**−**^
ABO blood group types		
AA	0.036784	0.011616
AO	0.207328	0.065472
BB	0.019456	0.006144
BO	0.150784	0.047616
AB	0.053504	0.016896
OO	0.292144	0.092256

## Data Availability

The data used in this study will be available from the corresponding author upon reasonable request.
